# Patient and provider knowledge of and attitudes toward medical conditions and medication during pregnancy

**DOI:** 10.1186/s13722-021-00228-8

**Published:** 2021-03-29

**Authors:** Caroline Shadowen, Rachel Wheeler, Mishka Terplan

**Affiliations:** 1 Virginia Commonwealth University School of Medicine, 1201 East Marshall Street, #4-100, Richmond, VA 23298 USA; 2 Department of Obstetrics and Gynecology, VCU Hospital System, 1101 East Marshall Street, Sanger Hall, #11-022, Richmond, VA 23298 USA; 3 Friends Research Institute, 1040 Park Ave, Suite 103, Baltimore, MD 21201 USA; 4 National Clinician Consultation Center, University of California, San Francisco, USA

**Keywords:** Opioid use disorder, Medication, Knowledge, Attitudes, Medical education, Stigma

## Abstract

**Background:**

Knowledge of medical conditions and their evidence-based medications varies among individuals. This range of knowledge may affect attitudes and influence medical decision-making of both patients and providers. Perceptions may be even more impactful in pregnancy, a timeframe subject to bias, and in diseases that include behavioral symptoms and often carry significant societal stigma, such as opioid use disorder (OUD). We present our findings from a survey assessing participants’ knowledge of three distinct medical conditions (diabetes mellitus, bipolar disorder, and OUD) and how this knowledge affects perceptions of these disease states during pregnancy.

**Methods:**

Using existing surveys in the literature as a guideline, we designed a cross-sectional survey including multiple-choice questions to evaluate our hypothesis that less knowledge about a medical condition would result in more negative opinions towards that condition and its treatment throughout pregnancy. Participants responded to perception statements using a 5-point Likert scale (1 = “strongly disagree,” 5 = “strongly agree”). Surveys were administered to patients in prenatal care, patients in OUD treatment, medical students, and medical residents within a single institution. Response means were generated and compared using t tests and ANOVA.

**Results:**

A total of 323 participants completed the survey. There were differences in knowledge between respondent groups and by disease state, with prenatal patients having the least knowledge of all groups about OUD diagnosis (88.5% of prenatal patients answered correctly) and its treatment (91.8% answered correctly). Overall Likert means of all responses demonstrated that participants agreed that new mothers with OUD (mean 4.27, 5 = “strongly agree”) and their babies (4.12) would have challenges that others would not, compared to mothers with bipolar disorder (4.03) and their babies (3.60) as well as mothers with diabetes (3.87) and their babies (3.47), p < .001. Overall, respondents were likely to agree that women with OUD should not try to get pregnant (3.47), whereas they overall disagreed with that statement when it pertained to women with bipolar disorder (2.69, 2 = “disagree”) or diabetes (2.12), p = 0.03.

**Conclusions:**

With this single-center study, we found that, though there were gaps in knowledge regarding disease and disease treatment during pregnancy, less knowledge was not associated with more negative perceptions of disease and disease treatment during pregnancy. Perceptions were especially negative toward pregnant women with OUD. Increasing awareness of lived experiences of patients with disease, as well as the biases carried by both patients and providers, could improve treatment of chronic diseases and outcomes for patients.

**Supplementary Information:**

The online version contains supplementary material available at 10.1186/s13722-021-00228-8.

## Background

Pregnancy is a unique period of the female life course, one during which most women experience increased contact with the healthcare system [1]. This presents an opportunity for patients to receive not only prenatal care but also treatment for chronic conditions. In addition to being a time of physical and physiological change, pregnancy is often a time of increased motivation for lifestyle and behavioral change [2]. Healthcare providers who care for women during pregnancy frequently use the health of the fetus and the patient’s emerging identity as a mother to support and motivate positive change [3].

Chronic illness such as diabetes, migraines, and hypertension are common, and medication management in pregnancy, including dose adjustments or preferred formulations to minimize risk to the developing fetus, well-established [4, 5]. Most pregnant women with chronic medical conditions receive treatment during pregnancy. Behavioral health conditions such as depression, bipolar disorder, and opioid use disorder (OUD), are also common and common in pregnancy. Although medication management for these diseases is also well-established, receipt of care lags; less than half of women with any mental illness receive mental health services [6]. Less than 20% of pregnant women receive any treatment for OUD [7] despite opioid use and use disorder being more common among women than men, with continued rising rates and increasingly affecting pregnancies [8, 9].

There is a two-fold disconnect between those affected by these disorders and the health care providers who treat them [10]. First, there is a lack of knowledge when it comes to how mental illness or OUD intersect with women’s health, particularly in how these conditions and their respective medication treatments may affect pregnancy [11]. Second many patients and practitioners maintain strong opinions—often negative ones—toward individuals with psychiatric and substance use disorders [12]. Lack of knowledge and inherent biases can have negative effects on the type of care a patient receives. This is even more pronounced in vulnerable populations who may carry strong self-stigma, such as those in mental health or substance use treatment [13]. Many of these patients also carry stigma placed upon them by care providers from whom they’ve previously received treatment [14].

Appropriately, there are many concerns surrounding medications during pregnancy, including potential side effects to a developing fetus, dose adjustments required due to the physiologic changes in pregnancy, and risk–benefit analysis to cause least harm to the maternal–fetal dyad. Although research typically focuses on the risk profile of specific medications for the developing fetus, many providers rely on their clinical gestalt in assessing risk and benefit, which often inadvertently takes into account their own personal experiences and biases [15].

Knowledge of various medical conditions and their respective medications can vary between individuals and can affect attitudes toward these conditions. These attitudes can, in turn, influence medical decision-making on the parts of either patients or providers. Data is limited on patients’ or providers’ attitudes toward medications prescribed and taken in pregnancy, especially pertaining to mental illness and substance use disorders.

This single-center study aimed to first evaluate patient, student, and resident knowledge of three conditions (type I diabetes, bipolar disorder, and OUD) and one of their respective, evidence-based medications. These conditions were chosen because medical guidelines recommend their medication initiation and continuation throughout pregnancy. The specific medications (insulin, lithium, and methadone) were chosen because they are commonly used to treat their respective conditions, both apart from and during pregnancy. Insulin, lithium, and methadone may present side effects to both mother and fetus, and current evidence supports the use of shared decision-making for providers who are treating these chronic conditions to maximize outcomes and minimize adverse risks [16, 17].

Second, the study investigated participant groups’ attitudes towards women with those diseases during pregnancy, including their use of the aforementioned medications. We hypothesized that less knowledge about a medical condition would result in more negative opinions towards that condition and its treatment throughout pregnancy. Further, we hypothesized that participants would know less about OUD due to negative perceptions of this disorder on a societal level and subsequently express more negative attitudes toward women with those conditions and toward its appropriate medication being used for management during pregnancy.

## Methods

This study was approved by the Virginia Commonwealth University Institutional Review Board, reference #HM20013375. To design our survey, we first reviewed the current scientific literature for studies that included surveys investigating knowledge and perceptions of disease states. Utilizing these existing instruments as a guide [12, 18, 19], we designed a survey to assess several domains pertaining to our research questions.

The first section of the survey inquired about basic demographic information, including gender identity and lived participant experience (e.g., if respondent has ever been pregnant or has a diagnosis of diabetes). This brief demographics section was included to assess any baseline differences between participants, as well as to gather information about participant categorization to investigate any associations between lived experience and perceptions.

The next section of the survey assessed participants’ knowledge of three medical conditions—diabetes mellitus type I, bipolar disorder, and OUD—and one of their respective evidence-based medications—insulin, lithium, and methadone. We chose these medications for each disease because they are commonly-used treatments which current guidelines advise may be continued throughout pregnancy. The knowledge section of the survey was included to assess participants’ baseline understanding of the three medical conditions and their treatments and to allow the research team to investigate any differences in knowledge between diseases and between participant groups.

The last section of the survey asked participants to respond to a series of statements regarding their attitudes toward the three disorders and their medications. This section was included to assess perceptions of these diseases and their medications as used in the general patient population, as well as their use specifically in pregnant patients. Participants responded to perception statements using a 5-point Likert scale (1 = strongly disagree, 2 = disagree, 3 = neutral, 4 = agree, 5 = strongly agree). An example of a statement that illustrated negative perceptions is “A woman with X disorder should not try to get pregnant.” Full text of the survey is available in (Additional file 1).

The survey was administered to four separate groups at a single institution: patients in prenatal care, patients in OUD treatment, medical students, and residents. We chose to distribute surveys to residents in Obstetrics & Gynecology, Emergency Medicine, and Pediatrics and not to residents in other specialties because of these residents’ close interaction with prenatal patients, patients with OUD, and neonates born to mothers with OUD, given the survey’s focus on OUD and pregnancy. Patients were approached in waiting rooms of relevant clinics where research staff were approved. All medical students at our institution were sent the survey via email. Residents were approached to complete the survey at weekly didactic sessions and during downtime on service. Because of the myriad methods used to capture as many responses as possible from all four groups, we were not able to calculate a response rate.

Patients, students, and residents who were under the age of 18 or were not proficient enough in English to comprehend a survey were excluded. Surveys were completed in paper form in-person by patients and residents, and administered online via RedCap (survey administration software used at our institution) to medical students.

Basic demographic data were compared using Fischer exact tests. Knowledge and attitudes were reported as Likert scores and means analyzed with ANOVA. We tested our primary hypothesis by comparing knowledge assessment (defined as those who scored 100% on the knowledge test) by mean Likert of the opioid attitudes. Stata (v13) was used for all analysis.

## Results

### Demographics

A total of 323 individuals completed the survey: 99 patients in prenatal care, 61 patients in OUD treatment, 99 medical students, and 64 residents. Demographics information from the survey demonstrated that the four groups differed in terms of lived experience of pregnancy, diagnosis of bipolar disorder, and diagnosis of OUD. The four groups were similar only in terms of lived experience of diabetes (Table 1).

**Table 1:** Demographic characteristics of study population

	Prenatal patients n (%)	Patients with OUD n (%)	Medical students n (%)	Residents n (%)	Overall n (%)	P-Value
Gender						
Female	99 (100)	29 (47.5)	55 (55.6)	28 (43.8)	210 (65)	< 0.001
Male	0 (0)	29 (47.5)	44 (44.4)	36 (56.3)	110 (34)	
Other	0 (0)	3 (4.9)	0 (0)	0 (0)	3 (0.9)	
Personal history of pregnancy	74 (76.3)	26 (44.1)	2 (2.0)	3 (4.7)	105 (32.0)	< 0.001
Personal diagnosis of diabetes	2 (2.0)	2 (3.3)	2 (2.0)	2 (3.1)	8 (2.5)	0.83
Personal diagnosis of bipolar disorder	7 (7.1)	19 (31.2)	1 (1.0)	0 (0)	27 (8.3)	< 0.001
Personal diagnosis of OUD	2 (2.0)	50 (82.0)	0 (0)	0 (0)	52 (16.2)	< 0.001

### Knowledge

The four groups differed in their knowledge of the diagnostic criteria and appropriate medication for each of the three medical conditions (Table 2). For OUD knowledge questions, the results between the four groups differed (p < 0.001 for both diagnosis and medication questions). Patients with OUD performed almost as well as residents and medical students, with 96.5% correctly answering the question of diagnosis, compared to 98.4% of residents and 100% of medical students, and 98.3% correctly identifying methadone as a treatment for OUD, compared to 100% of residents and 99.0% of medical students.

**Table 2:** Knowledge of medical condition and medication management by participant group

	Prenatal patients n (%)	Patients with OUD n (%)	Medical students n (%)	Residents n (%)	Overall n (%)	P-value
Correctly answered question about diabetes	66 (75.0)	34 (63.0)	99 (100)	64 (100)	263 (86.2)	< 0.001
Correctly answered question about diabetes medication	90 (97.8)	52 (94.6)	99 (100)	64 (100)	305 (98.4)	0.03
Correctly answered question about bipolar	80 (86.0)	53 (93.0)	98 (99.0)	64 (100)	295 (94.3)	< 0.001
Correctly answered question about bipolar medication	48 (58.5)	44 (83.0)	93 (94)	64 (100)	249 (83.6)	< 0.001
Correctly answered question about OUD	77 (88.5)	55 (96.5)	99 (100)	63 (98.4)	294 (95.8)	< 0.001
Correctly answered question about OUD medication	78 (91.8)	57 (98.3)	98 (99.0)	64 (100)	297 (97.1)	0.01

### Perceptions

For perception statements, we compared mean Likert responses by group (prenatal patients, patients with OUD, medical students, and residents) and by domain for each of the disease states (Table 3). We also compared overall means to evaluate differences in responses based on disease state.

**Table 3:** Assessment of attitudes toward statements about medical conditions and medications during pregnancy by participant group

Group (n)	Statement	Mean likert of response (standard deviation)			P-value
		Diabetes	Bipolar	OUD	
Overall (323)	Medication is important part of treatment	4.51 (0.77)	3.92 (0.85)	4.02 (0.91)	< 0.001
	Pregnant woman should take medication if MD recommends	4.41 (0.81)	3.43 (1.13)	3.74 (1.02)	< 0.001
	Baby will have challenges	3.47 (1.02)	3.60 (1.05)	4.12 (0.92)	< 0.001
	New mother will have challenges	3.87 (0.90)	4.03 (0.96)	4.27 (0.69)	< 0.001
	Side effects of medication are harmful to fetus	2.84 (0.96)	3.83 (0.90)	3.46 (0.93)	< 0.001
	Woman with this condition should not try to get pregnant	2.12 (0.94)	2.69 (1.05)	3.47 (1.17)	0.03
	Woman should be tested throughout pregnancy to ensure compliance to medication	3.48 (1.03)	3.33 (1.00)	3.73 (0.92)	< 0.001
Prenatal patients (99)	Medication is important part of treatment	4.19 (0.86)	3.54 (1.00)	3.71 (0.99)	< 0.001
	Pregnant woman should take medication if MD recommends	4.10 (0.92)	3.46 (1.06)	3.40 (1.10)	0.01
	Baby will have challenges	3.20 (0.95)	3.14 (1.08)	3.79 (1.05)	< 0.001
	New mother will have challenges	3.58 (0.96)	3.74 (1.11)	3.98 (0.94)	0.003
	Side effects of medication are harmful to fetus	2.93 (0.92)	3.51 (0.78)	3.70 (0.88)	< 0.001
	Woman with this condition should not try to get pregnant	2.36 (0.94)	2.67 (1.00)	3.76 (1.09)	0.15
	Woman should be tested throughout pregnancy to ensure compliance to medication	3.57 (0.96)	3.53 (0.95)	3.87 (0.99)	< 0.001
Patients with OUD (61)	Medication is important part of treatment	4.13 (0.91)	3.64 (0.80)	3.76 (1.01)	0.01
	Pregnant woman should take medication if MD recommends	4.15 (0.94)	3.76 (0.89)	3.33 (1.24)	0.07
	Baby will have challenges	3.08 (1.06)	3.38 (1.02)	3.82 (1.14)	0.40
	New mother will have challenges	3.50 (1.01)	3.69 (1.07)	3.94 (0.90)	0.09
	Side effects of medication are harmful to fetus	3.00 (0.80)	3.30 (0.81)	3.72 (0.94)	0.38
	Woman with this condition should not try to get pregnant	2.71 (1.09)	3.12 (1.11)	4.02 (1.10)	0.10
	Woman should be tested throughout pregnancy to ensure compliance to medication	3.80 (0.96)	3.85 (0.74)	3.94 (0.74)	0.004
Medical students (99)	Medication is important part of treatment	4.87 (0.34)	4.28 (0.60)	4.31 (0.70)	0.19
	Pregnant woman should take medication if MD recommends	4.73 (0.50)	3.52 (1.25)	4.09 (0.69)	< 0.001
	Baby will have challenges	3.65 (1.00)	3.89 (0.97)	4.52 (0.69)	< 0.001
	New mother will have challenges	4.22 (0.63)	4.32 (0.73)	4.61 (0.57)	0.01
	Side effects of medication are harmful to fetus	2.69 (1.04)	3.95 (0.91)	3.29 (0.83)	0.06
	Woman with this condition should not try to get pregnant	1.87 (0.79)	2.63 (1.03)	3.48 (1.02)	0.21
	Woman should be tested throughout pregnancy to ensure compliance to medication	3.47 (1.07)	3.08 (1.02)	3.62 (0.85)	< 0.001
Residents (64)	Medication is important part of treatment	4.82 (0.39)	4.13 (0.65)	4.31 (0.69)	0.11
	Pregnant woman should take medication if MD recommends	4.67 (0.59)	3.02 (1.13)	4.08 (0.78)	0.03
	Baby will have challenges	3.94 (0.91)	4.03 (0.85)	4.27 (0.70)	< 0.001
	New mother will have challenges	4.06 (0.80)	4.33 (0.65)	4.46 (0.69)	< 0.001
	Side effects of medication are harmful to fetus	2.81 (0.98)	4.53 (0.56)	3.20 (0.99)	< 0.001
	Woman with this condition should not get pregnant	1.75 (0.72)	2.46 (1.01)	2.58 (1.05)	0.36
	Woman should be tested throughout pregnancy to ensure compliance to medication	3.19 (1.03)	3.03 (0.98)	3.52 (1.01)	0.28

Likert score means aggregated from the four groups demonstrated significant differences in perceptions between disease states (p < 0.001). Overall, participants agreed that new mothers with OUD (4.27, 5 = “strongly agree”, standard deviation (SD) 0.69) and their babies (4.12, SD 0.92) would have challenges that others would not, compared to mothers with bipolar disorder (4.03, SD 0.96) and their babies (3.60, SD 1.05), as well as mothers with diabetes (3.87, SD 0.90) and their babies (3.47, SD 1.02). There was also a significant difference between perceptions of women’s suitability for pregnancy based on disease state (p = 0.03): among all participants, more were likely to agree that women with OUD should not try to get pregnant (3.47, SD 1.17), whereas participants overall disagreed with that statement when it pertained to women with bipolar disorder (2.69, SD 1.05) or diabetes (2.12, SD 0.94).

Perception statements answered by prenatal patients followed the overall trend regarding unique challenges women may face; prenatal patients were more likely to agree that mothers with OUD would face unique challenges (3.98, SD 0.94) compared to mothers with bipolar disorder (3.74, SD 1.11) and diabetes (3.58, SD 0.96), p = 0.03. They were also more likely to agree that side effects for medication used to treat OUD were harmful to a developing fetus (3.70, SD 0.88) compared to medication for bipolar disorder (3.51, SD 0.78) or diabetes (2.93, SD 0.92), p < 0.001.

Patients in treatment for OUD were more likely to agree that new mothers with OUD (3.94, 4 = “agree”, SD 0.90) and babies born to them (3.82, SD 1.14) would have more challenges than new mothers with bipolar disorder (3.69, SD 1.07) and their babies (3.38, SD 1.02), as well as new mothers with diabetes (3.50, SD 1.01) and their babies (3.08, SD 1.06). Patients with OUD were less likely to agree that pregnant women should take medication for OUD if their doctor recommended it (3.33, SD 1.24) than medication for bipolar disorder (3.76, SD 0.89) or diabetes (4.15, SD 0.94). Patients in OUD treatment were more likely to agree with the statement that women with OUD should not try to get pregnant (4.02, with 4 being “agree”, SD 1.10) than the same statement about women with bipolar disorder (3.12, SD 1.11) and diabetes (2.71 with 2 being “disagree”, SD 1.09). Patients with OUD were the most likely of the four participant groups to agree that pregnant women with OUD should be tested throughout pregnancy to ensure adherence with medication (3.94, SD 0.74), and they were more likely to agree with this statement about women with OUD than women with bipolar disorder (3.85, SD 0.74) or diabetes (3.80, SD 0.96), p = 0.004.

Medical students were likely to agree that babies born to mothers with OUD would face challenges that others would not (4.61, 4 = “agree”, SD 0.57), compared to mothers with bipolar disorder (3.89, SD 0.97) and diabetes (3.65, SD 1.00), p < 0.001. Medical students slightly agreed with the statement that women with OUD should not try to get pregnant (3.48, 3 = “neutral”, SD 1.02), whereas they overall disagreed with that statement when asked about women with bipolar disorder (2.63, SD 1.03) or diabetes (1.87, 1 = “strongly disagree”, SD 0.79).

Residents agreed that mothers with OUD (4.46, SD 0.69) and their babies (4.27, SD 0.70) would face challenges, compared with mothers with bipolar disorder (4.33, SD 0.65) and their babies (4.03, SD 0.85) or mothers with diabetes (4.06, SD 0.80) and their babies (3.94, SD 0.91). Similarly, residents disagreed less strongly with the statement that women with OUD should not try to get pregnant (2.58, with 2 being “disagree”, SD 1.05), compared with the same statement about women with bipolar disorder (2.46, SD 1.01) and diabetes (1.75, SD 0.72), p < 0.001.

Table 4 assesses the relationship between OUD knowledge and mean Likert responses related to OUD attitudes. Overall, individuals who correctly answered knowledge questions about OUD were slightly more likely to endorse statements that both the new mother (4.32, SD 0.80) and her new baby (4.19, SD 0.91) will have challenges than those individuals who did not answer the OUD knowledge questions correctly (p = 0.01 for mother, p < 0.001 for baby).

**Table 4:** Association between OUD knowledge and attitudes toward statements pertaining to OUD

Statement	Mean Likert (standard deviation)		P-Value
	Knowledge Correct^a^	Knowledge Incorrect^b^	
Medication is important part of treatment	4.06 (0.91)	3.54 (0.78)	0.06
Pregnant woman should take medication if MD recommends	3.78 (1.01)	3.33 (1.07)	0.07
Baby will have challenges	4.19 (0.91)	3.08 (1.24)	< 0.001
New mother will have challenges	4.32 (0.80)	3.58 (1.08)	0.01
Side effects of medication are harmful to fetus	3.45 (0.91)	3.50 (1.45)	0.02
Woman with OUD should not try to get pregnant	3.48 (1.17)	3.00 (1.35)	0.34
Woman should be tested throughout pregnancy to ensure compliance to medication	3.72 (0.90)	3.82 (1.17)	0.25

^a^Participants who selected the correct answer from the multiple-choice knowledge questions regarding diagnosis and treatment of OUD

^a^Participants who did not select the correct answer from the multiple-choice knowledge questions regarding diagnosis and treatment of OUD

## Discussion

Perceptions toward disease and disease treatment can impact medical decision-making, especially in pregnancy, a timeframe particularly subject to bias. Our study evaluated four groups’ knowledge of three distinct diseases and their respective medications, as well as perceptions of how those diseases and medications interact with pregnancy. Across all participants, there were more negative perceptions toward OUD in pregnancy, such as the challenges that mothers with OUD may face and the decision of women with OUD to seek pregnancy. We anticipated these negative perceptions toward OUD but did not find our hypothesized association between less knowledge and more negative opinion. Based on our study findings, we advocate for a nuanced approach to stigma reduction for OUD in pregnancy, to include education as well as awareness of personal bias and exposure to those with lived experience when possible.

Our findings did not demonstrate our hypothesized association between knowledge of OUD diagnosis/treatment with perceptions of the disorder: less knowledge did not increase the likelihood of negative perceptions. Overall, our results demonstrated mixed directionality, and in fact, for some indicators, increased knowledge was associated with increased negative perceptions towards new mothers with OUD and their babies. This finding could be attributed in part to the overall high percentage of correct knowledge answers regarding OUD. It could also be related to the fact that lack of knowledge alone is insufficient to explain biases; despite having knowledge of a disease, people may still hold discriminatory beliefs about that disease and those who live with it [20]. Our findings speak to the limitations of education as a vehicle to change perception and, consequently, behavior. Studies on stigma demonstrate that exposure to people with lived experience, either through narrative or direct contact, can be more effective in decreasing biases than interventions that aim only to educate [21]. To combat stigma and discrimination for both learners and patients, we need strategies to be more than educational and perhaps include narrative exposure and direct contact with patients with lived experience.

In our study, knowledge was assessed using a series of multiple-choice questions that asked basic questions about diagnosis and medical management of diabetes, bipolar disorder, and OUD. Overall, participants knew the least about diagnosis of diabetes and medication for bipolar disorder. We postulate that this could be due to low numbers of lived experience of these diagnoses among the participants. Further highlighting the impact of lived experience on knowledge, patients in treatment for OUD performed significantly better on questions pertaining to OUD diagnosis and medication compared with prenatal patients, who were much less likely to report lived experience with OUD. Personal experience with a disease process can positively impact one’s perception of a disease; conversely, lack of personal experience often contributes to negative bias toward a condition and the people who suffer from it [22]. Though overall the percentage of correct responses to knowledge questions was high, there were still gaps in knowledge for certain indicators, especially from patients, both those in prenatal care and those in OUD treatment. Based on our findings, we advocate for more education generally for patients when seeking medical care, particularly universal education for prenatal patients on medical conditions that may affect pregnancy.

All four participant groups were more likely to agree with the statement “Women with OUD should not try to get pregnant” than with the same statement about diabetes or bipolar disorder. Interestingly, patients in treatment for OUD were the most likely of all four groups to agree with this statement, possibly demonstrating self-stigma and/or personal understanding of the consequences of the disease on pregnancy and motherhood. Self-stigma can play a significant role in the disease trajectory of OUD [23]. Absorbing public perceptions of the disease can impact how individuals with OUD seek medical help, maintain medication treatment, and participate in behavioral treatment [24]. For pregnant women, for whom stigma surrounding substance use can be even greater than their non-pregnant counterparts, lack of quality healthcare due to self-stigma and shame can impact both maternal and fetal outcomes [25]. Our study indicates that patients who are being treated for OUD are adequately knowledgeable about the basic diagnosis and treatment of the disorder. However, our findings may demonstrate a potential need for improved efforts to specifically target self-stigma, such as peer support groups and individual behavioral therapy, to empower these patients. Further research may aid in investigating other meaningful ways to improve self-perception for patients with OUD.

We found that medical students and residents were more likely to agree that women with OUD should be tested throughout pregnancy to ensure medication compliance than women with bipolar disorder or diabetes. This indicates potential bias that providers are bringing into their interactions with patients with OUD, an inherent mistrust and perception that these patients will not do what is asked of them compared to other patients. This negative bias can affect clinical care and outcomes. Providing a stigma-free (or, at least, stigma-aware and stigma-reduced) clinical environment is critical to properly address OUD in pregnancy [26, 27]. Our survey’s knowledge questions demonstrated that medical students and residents are adequately knowledgeable about the basic diagnosis and treatment of the disorder. However, further work needs to be done to address biases that exist both within medical education and in medical culture at large; such biases influence the ways providers interact with patients in OUD treatment and choices providers make while treating this disease.

There are several limitations to this study. First, the survey was distributed at a single institution and may not be generalizable to other environments. However, our institution serves a patient population of diverse cultural, ethnic, and socioeconomic backgrounds, which may be a strength of our study. Another limitation is that our survey did not ask specifically for participants to directly compare the disease conditions in terms of their perceived effects on pregnancy; rather, we extrapolated a comparison of attitudes from the individual questions. While the indirect statements are a reasonable method to compare attitudes, a direct comparison question could have provided a more targeted assessment. Additionally, the patients in treatment for OUD whom we surveyed were being treated at a clinic that exclusively prescribes buprenorphine. However, the survey inquires specifically about methadone for the treatment of OUD. If we were interested in more directly correlating lived experience with knowledge and attitudes, we could have considered using buprenorphine as the medical treatment for OUD in all survey questions regarding treatment of OUD instead of methadone.

## Conclusions

We did not find sufficient evidence from this survey to support our hypothesis that less knowledge about a medical condition would result in more negative opinions towards that condition and its treatment throughout pregnancy. We did find that knowledge and attitudes toward diseases and treatments during pregnancy differed both by disease condition and between participant groups of medical trainees and patients. Overall negative statements were endorsed more for OUD than for diabetes or bipolar disorder, possibly illustrating how stigma for OUD or concern about pregnant patients with OUD is greater than for other health conditions. Pregnant women with chronic health conditions, especially OUD, face stigma, which can result from lack of evidence-based information, as well as patient- or provider-held beliefs about what is best for a developing fetus or new mother. Medical providers need to be aware of knowledge gaps that their patients may have, as well as the biases that they themselves bring into interactions with pregnant patients. This awareness is particularly important with patients in more vulnerable, stigmatized populations like patients with OUD.

## Supplementary Information

**Additional file 1.** Survey questionnaire.

## Data Availability

The datasets used and/or analysed during the current study are available from the corresponding author on reasonable request.
