# Supplementary material for: Patient and provider knowledge of and attitudes toward medical conditions and medication during pregnancy
Source: Addict Sci Clin Pract. 2021 Mar 29;16:22. doi: 10.1186/s13722-021-00228-8 (PMC8008637; doi:10.1186/s13722-021-00228-8)
Supplement: Supplementary file 1 — Additional file 1. Survey questionnaire. [file 13722_2021_228_MOESM1_ESM.docx]

Survey Questionnaire

***Knowledge Questions***:

1. A diagnosis of diabetes type I means that:

**O A.** a person’s heart does not pump enough blood to the rest of the organs.

**O B.** a person’s body does not make insulin.

**O C.** a person’s blood vessels are blocked.

**O D**. a person’s body does not make enough sugar.

1. A commonly used medication in treating diabetes type I is:

**O A.** Tylenol

**O B.** Penicillin

**O C**. Insulin

**O** **D.** Albuterol

1. A diagnosis of bipolar disorder means that:

**O A**. a person’s body does not grow hair.

**O B.** a person experiences anxious mood states.

**O C**. a person experiences periods of elevated mood (mania) and depression.

**O D**. a person’s body is asymmetrical.

1. A commonly used medication in treating bipolar disorder is:

**O A**. Lithium

**O B.** Aspirin

**O C.** Propranolol

**O D**. Epinephrine

1. A diagnosis of opioid use disorder means that:

**O A**. a person is an alcoholic.

**O B.** a person’s brain does not get enough blood from the heart.

**O C**. a person’s body does not build muscle.

**O D**. a person misuses a class of substances.

1. A commonly used medication in treating opioid use disorder is:

**O A.** Advil

**O B.** Methadone

**O C.** Claritin

**O D.** Ritalin

***Demographic Questions:***

1. Please select the group(s) to which you belong:

**O** medical student **O** woman who is pregnant

**O** patient at MOTIVATE **O** provider

1. If you selected “provider” above, please select the group to which you belong:

**O** resident **O** attending

1. If you selected “provider” above, please select the specialty you practice:

**O** Ob/Gyn **O** Emergency **O** Anesthesia

**O** Pediatrics **O** Internal Medicine **O** Psychiatry

1. Please select the gender with which you identify:

**O** male **O** female **O** other

1. Have you ever been pregnant?

**O** yes **O** no

1. Do you have a diagnosis of diabetes type I?

**O** yes **O** no

1. Do you have a diagnosis of bipolar disorder?

**O** yes **O** no

1. Do you have a diagnosis of opioid use disorder?

**O** yes **O** no

***Background Information:***

For the purposes of this survey, we will use the following diagnostic criteria based on current medical literature:

- **Diabetes type I:** an individual who is not able to make insulin on their own.

- **Bipolar disorder:** an individual who has episodes of mania (elevated mood) and major depression.

- **Opioid use disorder**: an individual who misuses prescribed opioid medication (Oxycodone, Percocet, etc) or illegal opioid substances (heroin). Commonly referred to as ***“opioid addiction.”***

***Opinion Questions:***

Please indicate the extent to which you agree with each statement below by marking the circle corresponding with your response.

There are no right or wrong answers, just your perceptions.

|  | Strongly Disagree | Disagree | Neutral | Agree | Strongly Agree |
| --- | --- | --- | --- | --- | --- |
| 1. Methadone is an important part of treatment for opioid use disorder. | **O** | **O** | **O** | **O** | **O** |
| 2. A person with opioid use disorder should take methadone if their doctor recommends it. | **O** | **O** | **O** | **O** | **O** |
| 3. A woman with opioid use disorder who is pregnant should take methadone if her doctor recommends it. | **O** | **O** | **O** | **O** | **O** |
| 4. A baby born to a mother with opioid use disorder will have challenges that another baby will not. | **O** | **O** | **O** | **O** | **O** |
| 5. A new mother with opioid use disorder will have challenges that a new mother without it will not. | **O** | **O** | **O** | **O** | **O** |
| 6. There are side effects to methadone that are harmful to a developing fetus. | **O** | **O** | **O** | **O** | **O** |
| 7. A woman with opioid use disorder should not try to get pregnant. | **O** | **O** | **O** | **O** | **O** |
| 8. A woman with opioid use disorder who is pregnant should be tested throughout her pregnancy to make sure she is taking her methadone. | **O** | **O** | **O** | **O** | **O** |
| 9. Lithium is an important part of treatment for bipolar disorder. | **O** | **O** | **O** | **O** | **O** |
| 10. A person with bipolar disorder should take lithium if their doctor recommends it. | **O** | **O** | **O** | **O** | **O** |
| 11. A woman with bipolar disorder who is pregnant should take lithium if her doctor recommends it. | **O** | **O** | **O** | **O** | **O** |
| 12. A baby born to a mother with bipolar disorder will have challenges that another baby will not. | **O** | **O** | **O** | **O** | **O** |
| 13. A new mother with bipolar disorder will have challenges that a new mother without it will not. | **O** | **O** | **O** | **O** | **O** |
| 14. There are side effects to lithium that are harmful to a developing fetus. | **O** | **O** | **O** | **O** | **O** |
| 15. A woman with bipolar disorder should not try to get pregnant. | **O** | **O** | **O** | **O** | **O** |
| 16. A woman with bipolar disorder who is pregnant should be tested throughout her pregnancy to make sure she is taking her lithium. | **O** | **O** | **O** | **O** | **O** |
| 17. Insulin is an important part of treatment for diabetes type I. | **O** | **O** | **O** | **O** | **O** |
| 18. A person with diabetes type I should take insulin if their doctor recommends it. | **O** | **O** | **O** | **O** | **O** |
| 19. A woman with diabetes type I who is pregnant should take insulin if her doctor recommends it. | **O** | **O** | **O** | **O** | **O** |
| 20. A baby born to a mother with diabetes type I will have challenges that another baby will not. | **O** | **O** | **O** | **O** | **O** |
|  | Strongly Disagree | Disagree | Neutral | Agree | Strongly Agree |
| 21. A new mother with diabetes type I will have challenges that a new mother without it will not. | **O** | **O** | **O** | **O** | **O** |
| 22. There are side effects to insulin that are harmful to a developing fetus. | **O** | **O** | **O** | **O** | **O** |
| 23. A woman with diabetes type I should not try to get pregnant. | **O** | **O** | **O** | **O** | **O** |
| 24. A woman with diabetes type I who is pregnant should be tested throughout her pregnancy to make sure she is taking her insulin. | **O** | **O** | **O** | **O** | **O** |
| 25. It is more important to consider the health of a woman who is pregnant than the developing fetus. | **O** | **O** | **O** | **O** | **O** |
| 26. It is more important to consider the health of a developing fetus than the health of the woman who is pregnant. | **O** | **O** | **O** | **O** | **O** |
